# Co-infection of two reoviruses increases both viruses accumulation in rice by up-regulating of viroplasm components and movement proteins bilaterally and RNA silencing suppressor unilaterally

**DOI:** 10.1186/s12985-017-0819-0

**Published:** 2017-08-08

**Authors:** Shu Li, Tong Zhang, Yingzhi Zhu, Guohui Zhou

**Affiliations:** Key Laboratory of Microbial Signals and Disease Control of Guangdong Province, College of Agriculture, South China Agricultural University, No 483 Wushan Road, Tianhe District, Guangzhou, Guangdong 510642 China

**Keywords:** Southern rice black-streaked dwarf virus, Rice ragged stunt virus, Co-infection, Synergism

## Abstract

**Background:**

Synergism between southern rice black-streaked dwarf virus (SRBSDV) and rice ragged stunt virus (RRSV) not only aggravates disease symptoms but also enhances their vector acquisition efficiencies by increasing both viruses’ titers in co-infected rice plants, which may exacerbate the epidemic of both viruses and cause significant damage to rice production. The molecular mechanism of viral synergism of these two viruses remains unexplored.

**Methods:**

Single and double infection of SRBSDV and RRSV were obtained with the viruliferous white-backed planthopper and brown planthopper inoculation on four-leaf stage rice seedlings, respectively, under experimental condition. The second upper leaf from each inoculated rice plants were collected at 9, 15, and 20 days post inoculation (dpi) and used for relative quantification of 13 SRBSDV genes and 11 RRSV genes by the reverse-transcription quantitative PCR. Viral gene expression levels were compared between singly and doubly infected samples at the same stage.

**Results:**

The movement protein and viroplasm matrix-related genes as well as the structural (capsid) protein genes of both viruses were remarkably up-regulated at different time points in the co-infected rice plants compared with the samples singly infected with SRBSDV or RRSV, however, the RNA silencing suppressor (P6) of only RRSV, but not of both the viruses, was up-regulated.

**Conclusions:**

The SRBSDV-RRSV synergism promoted replication and movement of both viruses and inhibited the host immunity by enhancing the gene suppressing effect exerted by one of them (RRSV).

**Electronic supplementary material:**

The online version of this article (doi:10.1186/s12985-017-0819-0) contains supplementary material, which is available to authorized users.

## Background

*Southern rice black-streaked dwarf virus* (SRBSDV) and *Rice ragged stunt virus* (RRSV) are members of *Reoviridae* family. SRBSDV is a novel species in genus *Fijivirus* and transmitted by the white-backed plant hopper (WBPH, *Sogatella furcifera*), while RRSV belongs to the genus *Oryzavirus* and is vectored by the brown plant hopper (BPH, *Nilaparvata lugens*); both vectors, the two major long-distance migratory rice pests in south and southeast Asia, transmit these viruses in a persistent, circulative-propagative manner [1, 2]. SRBSDV was first discovered in China in 2001 and was identified in 2008 [3], and has become one of most important disease in southern china, northern Vietnam and Japan in recent years [4, 5]. RRSV was first described in Indonesia in 1977 [1] and reported in China in 1978 with only scattered distribution and occasional outbreaks [6]. However, RRSV occurrence became prominent recently with the co-infection of SRBSDV, probably due to elevated vector acquisition efficiency induced by the synergism of these two viruses [7].

The SRBSDV genome has ten linear double-stranded RNA (dsRNA) segments containing 13 open reading frames (ORFs), which putatively encode six structural proteins (P1 to P4, P8 and P10) and seven non-structural proteins (P5-1, P5-2, P6, P7-1, P7-2, P9-1 and P9-2). Based on the results analyzed by bioinformatics, the P1 is an RNA-dependent RNA polymerase (RdRP), P2 is a major core structural protein, P3 is an outer shell B-spike protein, P4 is a capping protein, P8 is a minor core protein and P10 is a major outer-capsid protein [8]. P6 was identified as a viral RNA silencing suppressor (RSS) [9] and might participate in viroplasm formation with P5-1 and P9-1 [10, 11]. P7-1 is the major component of the tubules and probably a viral movement protein (MP) [12]. The functions of P5-2, P7-2 and P9-2 remain unknown.

The RRSV genome contains ten dsRNA segments with 11 ORFs putatively encoding eight structural proteins (P1 to P3, P4a, P5, P8 and P9) and three non-structural proteins (P6, P7 and P10). Among the structural proteins, P1 is a major core structural protein and P2 has been reported to be a putative guanylyl transferase [13]. P3 is a capsid shell protein and P4a putatively functions as an RdRp [14]. P5 appears to be a guanyltransferase associated with 5′-capping in the processes of viral replication and transcription [15]. P8 has been identified as a major outer-capsid protein [16, 17]. P9 contributes as a spike protein refer to transmission by the insect vector [18]. Among the non-structural proteins of RRSV, P6 functions as a viral RSS and a MP [19], P7 is a NTP-binding protein [20] and P10 is a component of viroplasm [21]. The in vivo production and possible function of P4b is yet to be determined.

Our previous report found that synergism between SRBSDV and RRSV increased both virus titers in co-infected rice plants [7]. However, the molecular mechanism of viral synergism of the two viruses remains unexplored. In this study, we compared the expression levels of the viral genes between the rice plants singly infected with SRBSDV or RRSV and those with co-infection of both viruses at different disease stages using the reverse-transcription quantitative polymerase chain reaction (RT-qPCR). We found that compared with rice plants infected by SRBSDV or RRSV individually, the expression levels of some genes involved in SRBSDV and RRSV viroplasm formation were significantly up-regulated, and yet, of the RSSs of the two viruses, only P6 of RRSV was up-regulated, while some of the structural genes of both viruses were up-regulated. These findings suggest that synergism of SRBSDV and RRSV stimulates replication and movement of both viruses and weakens the host immunity through the RSS function of one of them, which result in an increase in both viruses’ titers in doubly infected rice plants.

## Methods

### Test plants, viruses, and insects

The seeds of a rice cultivar ‘Qiuyou-998’ used in this research were maintained in our laboratory. The seeds were germinated by soaking in warm water, then sown in a plastic box sized 50 cm long, 25 cm wide and 8 cm high. The seedlings with uniform growth were individually transplanted into a culture tube half-filled with the nutrient solution culture (pH 4.5 ~ 5.0) when they were in the four-leaf stage [22]. The seedlings were allowed to grow in an incubator under the conditions of 28 °C and 16 h light/8 h dark.

The isolates of SRBSDV and RRSV were collected from diseased field in Guangzhou, Guangdong Province, China. Both isolates were transmitted to a number of Qiuyou-998 rice plants by WBPH and BPH, and grown in insect-proof greenhouses.

Five fourth-stage nymphs of WBPH or BPH, which breed on SRBSDV or RRSV infected rice plants for two or three generations, were collected and then moved to each rice seedling at four-leaf stage. SRBSDV or RRSV singly infected rice plants were derived from rice seedlings inoculated with viruliferous WBPHs or BPHs, respectively, SRBSDV and RRSV co-infected rice plants were derived from rice seedlings inoculated with both viruliferous insects. At least 30 rice seedlings were used in each inoculation. All the insects were removed manually after a 24-h exposure. Second upper leaf from each inoculated rice plants were collected at 9, 15, and 20 days after the 24-h insect exposure (days post inoculation, dpi), enclosed with aluminium foil paper, and put in a refrigerator of −70 °C after liquid nitrogen freezing. SRBSDV or/and RRSV were detected in all inoculated rice plants at 20 dpi by RT-PCR using a One Step RNA PCR kit (AMV) (TaKaRa, Dalian, China) and specific primers for SRBSDV and RRSV according to Wang et al. [23]. Rice plants postitive for SRBSDV or RRSV and both viruses were defined as singly and co-infected rice plants. In total, 12 plants from each infection treatment were used for viral gene expression experiments.

### RT- qPCR detection

Trizol RNAiso Plus (Invitrogen, Boston, MA) were used for total RNA extraction from frozen leaves from SRBSDV or RRSV singly or co-infected rice plants, and then treated with DNase I (TaKaRa). Spectrophotometry was used to determine the concentration and purity of the total RNA. Total RNA samples with A_260_/A_280_ and A_260_/A_230_ of 1.9 to 2.1 and greater than 2.0, respectively, can be used as templates for RT-qPCR.

Primer Express (version 3.0; Applied Biosystems, Foster City, CA) was used to design the RT-qPCR primers (Table 1) based on 13 and 11 genes of SRBSDV and RRSV, respectively. The U6 small nuclear RNA gene of rice was used as a housekeeper or control genes for RT-qPCR (Table 1).

**Table 1:** Reverse-transcription quantitative polymerase chain reaction primers used for quantification of southern rice black-streaked dwarf virus (SRBSDV) and rice ragged stunt virus (RRSV)

Purpose	Gene name	Target gene (GenBank accession no.)	Primer for RT-qPCR(5′-3′)	Size of expected amplicon (bp)
RRSV	RRSV P1	HM125559	AGGATCTCTTCTCAATGCAAGC	70
			AACTTCCACCTGGCAACGTC	
	RRSV P2	HM125560	GGCGTTCGATCTCGTGTTTAA	69
			GCGGGAAAATCATTGGCGTG	
	RRSV P3	AF020336	TCAAAGCTAGTGATGAGCGTCTGT	70
			CTGGCATAAAAACGAAGAGTCTGA	
	RRSV P4A	HM125562	GGCGTCAGTGTGTAGCAGCAT	66
			ATTGGGTAGAGTTGTTGTTTTGGAT	
	RRSV P4B	HM125562	CGCCATCCACAAAGCTATCA	63
			AACACTCTCGTAGCCTGCCAAT	
	RRSV P5	HM125562	CGCCATCCACAAAGCTATCA	59
			AACACTCTCGTAGCCTGCCAAT	
	RRSV S5	HM125543	GGCGGCATCGGGTTGT	59
			CACCTCTATCAAACGCAGTAACCA	
	RRSV S6	HM125554	GCTTTCGCGGTGCTCAA	59
			CTCCCAATTACGCACCGAAT	
	RRSV P7	HM125555	TGACGATTACGCCGAGACAAG	62
			CGGACGACTGGCCAATG	
	RRSV P8	HM125546	GGCTGAGCGTGCGGTTA	66
			TCAGCCTTGATATCGTTGTAGCA	
	RRSV P9	L79969	GGTGTGGCTCTACAACAAATGG	67
			ACGCCTCTTTCTCTGCTCCTCTA	
	RRSV P10	HM125568	CCGAGCCGCCATCATAGTA	63
			ACCTCAAAAACTCCAGACAGCATA	
SRBSDV	SRBSDV P1	JQ692572	TGTGATTTGAACTCAGAGACAGCTTTA	80
			TGACGTTGATAAGTTGATTGTTGAGA	
	SRBSDV P2	HM585278	GCGAACGGCTTTCTGCTTT	71
			GAATGTGCGAGTGATTTCATGAA	
	SRBSDVP3	HM585277	TCCCGGTGTTCGAGAAGTTC	64
			CAGCATTTTCCATCCAACCAT	
	SRBSDV P4	HM585276	TCAACGCTCGACAACCAAAA	72
			TTATCGTGATATCTGCTACGAATACCA	
	SRBSDV P5-1	HM585275	ATCAGATGATAACCTCAATCGTACCA	94
			TGAGCCAGTGAAGGTAATCATCATTA	
	SRBSDV P5-2		CGCTTAACAATTGTCGTGATATGAA	75
			GCGTGCTTCCGAAAAGTAAAGTT	
	SRBSDV P6	HM585274	AAGCAACAAACATCAACGTCAAA	69
			AACTACGTCGGCCCATGAAC	
	SRBSDV P7-1	HM58527	CCTAATGAAAACCCATCTACCTGTAAC	70
			CACGAATAAACATCAGCGACAAA	
	SRBSDV P7-2		CAAATATTTAGTAGAAGCAGAACGCAAGT	61
			ACATCTTTTTCGTCTTTCTCATACTCTTAA	
	SRBSDV P8	HM585272	GATTGTCTCCTTTGGATGATGTTG	72
			CCTGTCCTTCTTGAAATACACGTAAC	
	SRBSDV P9-1	HM585271	TGAAAGCGAATCCTCAACTAAAGA	68
			TTGAATCAGTGGAGGTGGGTTT	
	SRBSDV P9-2		CGAGATGAGCGATTCTGGTTT	70
			TTCGTGAATGATGTTGTTAACCTTTT	
	SRBSDV P10	EU784840	TCATCATTAGCGCGACTAGTTCA	64
			CGTCACTCGGCGTCGATAA	
Reference	U6	S83742	CGATAAAATTGGAACGATACAGA	72
			ATTTGGACCATTTCTCGATTTGT	

A PrimeScript RT reagent kit (TaKaRa) was used to reverse-transcribed the total RNA into cDNA. cDNA were synthesized using 2 μL of 5× RT buffer, 0.5 μL of RT Enzyme Mix I, 0.5 μL of oligo dT, 6 μL of RNase-free double-distilled (ddH_2_O), and 2 μg total RNA, the reaction was conducted at 42 °C for 15 min followed by 95 °C for 2 min. The qPCR was performed on a Cycler Dice Real Time System TP800 (TaKaRa) using 2 ng of cDNA, 0.5 μL each of the forward and reverse primers (10 μM), 12.5 μL of SYBR Premix Ex Taq II, and 9.5 of RNase-free ddH_2_O. The reactions were run for 95 °C for 30 s, followed by 40 cycles of 95 °C for 5 s and 60 °C for 30 s. No-template reaction that replacing cDNA with RNase-free ddH2O was used as negative control and the U6 small nuclear RNA gene of rice was used as a housekeeper or control genes for all detections. A 10-fold gradient dilution of cDNA was used to construct a dissolution curve and a relative standard curve for determining the specificity and amplification efficiency of each primer. All primers had a single peak for dissolution curve and exhibited 90% to 110% amplification efficiency in all reactions. Analysis of relative genes expression data using 2 − ΔΔCt method. At least six rice plants were used for each treatment, and each reaction was run in triplicate.

### Statistical analysis

The statistical software package SPSS 19.0 (IBM, Armonk, NY) was used to analyze the experimental data. A *t*-test with Bonferroni correction was used to compare gene relative expression levels in different treatment at the same stage.

## Results

### The expression levels of genes involved in the formation of viroplasms of both viruses were significantly up-regulated in SRBSDV and RRSV co-infected rice

**Fig. 1:**
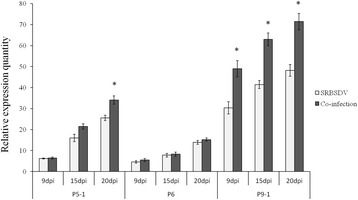
The expression levels of viroplasm-related genes of SRBSDV (P5-1, P6 and P9-1) at 9, 15 and 20 dpi in SRBSDV-infected and co-infected rice. Asterisks above vertical bars (with error bars indicating standard deviation) indicate significant differences in the expression levels of genes among differently infected rice plants on the indicated day post-infection (Bonferroni’s honestly significant difference test, *P* = 0.05)

The expression levels of three genes involved in the formation of SRBSDV viroplasms (P5-1, P6 and P9-1) were compared between SRBSDV singly infected and co-infected rice plants. No significant difference in the expression levels of P5-1 at 9 dpi was found between SRBSDV-infected rice plants and those co-infected with RRSV, but P5-1 was up-regulated 1.44 times at 15 dpi (*F* = 3.442, df = 6, *P* = 0.583) and 1.15 times at 20 dpi (*F* = 1.229, df = 6, *P* = 0.064) in co-infected plants. The expression of P9-1 was significantly up-regulated at all three tested disease progression time points; 1.62 times at 9 dpi (*F* = 2.307, df = 10, *P* = 0.003), 1.32 times at 15 dpi (*F* = 1.628, df = 8, *P* = 0.002) and 1.23 times at 20 dpi (*F* = 0.905, df = 10, *P* = 0.006). The expression levels of P6 showed no significant changes at any of the tested time points (Fig. 1).

**Fig. 2:**
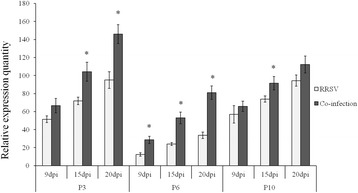
The expression levels of viroplasm-related genes of RRSV (P3, P6 and P10) at 9, 15 and 20 dpi in RRSV-infected and co-infected rice. Asterisks above vertical bars (with error bars indicating standard deviation) indicate significant differences in the expression levels of genes among differently infected rice plants on the indicated day post-infection (Bonferroni’s honestly significant difference test, *P* = 0.05)

The expression levels of three genes involved in viroplasm formation of RRSV (P3, P6 and P10) were compared in RRSV-infected and co-infected rice plants. The expression of P3 was up-regulated 1.29 times at 9 dpi (*F* = 0.342, df = 10, *P* = 0.592), 1.53 times at 15 dpi (*F* = 2.286, df = 8, *P* = 0.020) and 1.34 times at 20 dpi (*F* = 0.132, df = 8, *P* = 0.050). The expression of P6 was significantly up-regulated at all three tested disease progression time points; 1.43 times at 9 dpi (*F* = 0.456, df = 10, *P* = 0.018), 1.7 times at 15 dpi (*F* = 0.407, df = 10, *P* = 0.001), and 1.29 times at 20 dpi (*F* = 0.132, df = 10, *P* < 0.001). The expression level of P10 was also up-regulated at all three time points, 1.16 times at 9 dpi (*F* = 0.314, df = 10, *P* = 0.114), 1.25 times at 15 dpi (*F* = 1.026, df = 10, *P* < 0.000), and 1.19 times at 20 dpi (*F* = 0.004, df = 10, *P* = 0.098), in co-infected plants (Fig. 2).

### The expression level of viral RSS gene of RRSV was unilaterally up-regulated in SRBSDV and RRSV co-infected rice

The P6 proteins of SRBSDV and RRSV have been identified as the RSSs of these two viruses [12, 19]. No significant difference in the expression levels of SRBSDV P6 was found between SRBSDV-infected plants and those co-infected with RRSV at any of the tested disease progression time points (Fig. 1). However, the expression level of RRSV P6 was significantly up-regulated at 9, 15 and 20 dpi (Fig. 1).

### The expression levels of viral MP genes of both SRBSDV and RRSV were up-regulated in SRBSDV and RRSV co-infected rice

**Fig. 3:**
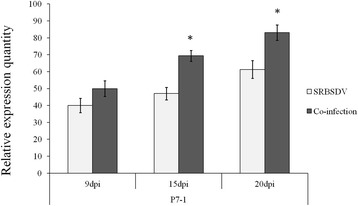
The expression levels of movement protein (MP) genes of SRBSDV (P7-1) at 9, 15 and 20 dpi in SRBSDV-infected and co-infected rice. Asterisks above vertical bars (with error bars indicating standard deviation) indicate significant differences in the expression levels of genes among differently infected rice plants on the indicated day post-infection (Bonferroni’s honestly significant difference test, *P* = 0.05)

P7-1 of SRBSDV was previously determined to be a viral MP [12]. It was up-regulated at all three tested time points in SRBSDV and RRSV co-infected plants. At 9 dpi, it was slightly up-regulated (*F* = 4.738, df = 10, *P* = 0.126), but at 15 dpi and 20 dpi, it was significantly up-regulated by 1.92 times (*F* = 1.937, df = 10, *P* = 0.010) and 1.23 times (*F* = 8.571, df = 10, *P* < 0.001), respectively, in co-infected rice compared with SRBSDV singly infected rice (Fig. 3). P6 of RRSV was also indentified as a viral MP [19], and it was significantly up-regulated at 9, 15 and 20 dpi in co-infected rice (Fig. 2).

### The expression levels of viral CPs of both SRBSDV and RRSV were unilaterally up-regulated in SRBSDV and RRSV co-infected rice

**Fig. 4:**
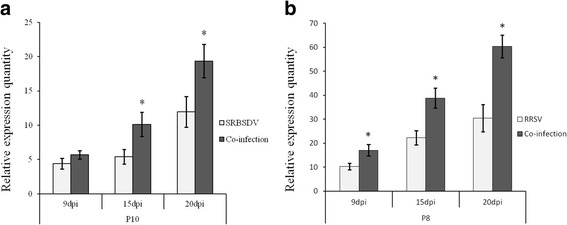
The expression levels of viral coat protein (CP) genes of SRBSDV (P10) (**a**) and RRSV (P8) (**b**) at 9, 15 and 20 dpi in SRBSDV- or RRSV-infected and co-infected rice. Asterisks above vertical bars (with error bars indicating standard deviation) indicate significant differences in the expression levels of genes among differently infected rice plants on the indicated day post-infection (Bonferroni’s honestly significant difference test, *P* = 0.05)

Compared with SRBSDV or RRSV single infection, the expression levels of all structural protein genes of both SRBSDV and RRSV were up-regulated to various degrees in co-infected plants (Additional file 1: Figure S1 and Additional file 2: Figure S2). Among them, changes in the expression levels of SRBSDV P10 and RRSV P8 reached statistically significant level (Fig. 4). SRBSDV P10 was slightly up-regulated at 9 dpi (*F* = 0.420, df = 10, *P* = 0.085) and significantly up-regulated 2.06 times at 15 dpi (*F* = 1.116, df = 10, *P* = 0.029) and 1.43 times at 20 dpi (*F* = 13.658, df = 10, *P* = 0.004) in co-infected plants. RRSV P8 was significantly up-regulated at all three tested disease progression time points, 1.44 times at 9 dpi (*F* = 0.005, df = 16, *P* = 0.040), 1.76 times at 15 dpi (*F* = 2.442, df = 26, *P* < 0.001), and 1.41 times at 20 dpi (*F* = 0.132, df = 8, *P* = 0.050), in co-infected plants (Fig. 4).

## Discussion

SRBSDV and RRSV, which are both in the family *Reoviridae*, have for the past few years caused serious rice disease in eastern and southeastern Asia. Our prior studies have shown that there is a synergistic interaction between SRBSDV and RRSV [7]. Rice plants co-infected with both viruses display aggravated symptoms, while the titers of both viruses and the acquisition efficiencies of both vectors are increased, which is of great epidemiological importance. However, there has not been sufficient research on the mechanism of synergistic interaction between plant viruses in the same family. Hence, we studied the expression levels of all genes of SRBSDV and RRSV at three representative stages of the disease course (9, 15 and 20 dpi), in order to expand our knowledge of the synergistic interaction between two viruses in the same family.

On the basis of previous research, SRBSDV is a dsRNA virus, 3′ end of the genome lacks poly-A tail [3]. In this research, the single-stranded cDNA templates of 13 genes of SRBSDV and 11 genes of RRSV were simultaneously synthesized using oligo-dT anchor primers according to He et al. [24], to avoid confusion between the virus genomes and transcripts.

The expression levels of P5-1, P7-1, P9-1 and P10 of SRBSDV and P3, P6, P8 and P10 of RRSV were significantly up-regulated in co-infected rice. These up-regulated genes include some genes associated with the formation of the viral factory (viroplasm) matrix (P5-1 and P9-1 of SRBSDV and P3, P6 and P10 of RRSV) [10, 11, 14, 19, 21], viral MPs (P7-1 of SRBSDV and P6 of RRSV) [12, 19] and viral CPs (P10 of SRBSDV and P8 of RRSV) [3, 17]. These up-regulated genes may play crucial roles in the synergism between SRBSDV and RRSV, and their upregulation also shows that co-infection increased both virus titers and facilitated the replication and movement of both viruses, which is consistent with our previous research [7].

RNA silencing functions as an antiviral defense response to virus genome expression, replication, and movement in plants, likewise, plant virus generally encode RSS to suppress host silencing that enable virus establish systemic infection in plants [25, 26]. Some research have shown that the function of viral RSSs often involved in synergism of plant virus [25–29]. Interestingly, the P6 proteins of SRBSDV and RRSV have been identified as the RSSs of these two viruses [9, 19], but only the expression level of protein P6 of RRSV was significantly up-regulated in co-infected plants. This unilateral influence mechanism is related to different silencing effects of the two viruses RSSs or effects in different nodes of the RNAi pathway. Thus, whether P6 of RRSV mediates the synergism between SRBSDV and RRSV and its possible mechanism deserve further study.

Compared with SRBSDV or RRSV single infection, most of the genes involved in viroplasm formation of SRBSDV and RRSV were significantly up-regulated in co-infected plants. Previous studies have shown that P6 of SRBSDV can interact with itself and P5-1, and might format the viroplasm by recruiting P9-1 [10, 11]. For RRSV, P10 is essential for viroplasm formation and virus replication; it can recruit P6 into viroplasm-like structures when these two proteins are co-expressed in Sf9 cells [21]. This study showed that the genes involved in viroplasm formation were up-regulated in co-infected plants; from another perspective, it proves that the replication levels of both viruses were enhanced. Importantly, however, the upregulation pattern of viroplasm-related genes varied in timing. At 9 dpi, only P9-1 of SRBSDV and P6 of RRSV were significantly up-regulated in co-infected plants, suggesting that these two genes might be the main limiting factors at the initial stage of viroplasm formation; viroplasms began to form only if the expression of these two genes reached a critical level. The expression of the other genes was significantly up-regulated at 15 or 20 dpi, suggesting that different genes become important at different stages of the formation and expansion of the viroplasm. In addition, the genes up-regulated at different times are likely to improve the interaction between the viroplasm-related genes, thereby enhancing the replication efficiency of the two viruses in co-infected plants.

Increasing amounts of experimental evidence show that, although MPs of different plant virus share lower sequence homology, they are functional commonalities. Consequently, the viral MPs of distinct species, and even distinct genus, can complement movement deficiency of each other and mediate the systemic movement and synergism of heterologous viruses [30–32]. P7-1 of SRBSDV and P6 of RRSV are recognized as viral MPs [12, 19]. These genes were significantly up-regulated at 9, 15 and 20 dpi in co-infected plants, which may facilitate the movement and spread of the two viruses, thereby exacerbating the symptoms of co-infected rice plants.

In this study, we found that the expression of viroplasm-related genes, the MP and CP genes were significantly up-regulated in the co-infected rice plants, but the expression levels of the other viral genes were not significantly affected by co-infection (Additional file 1: Figure S1, Additional file 2: Figure S2, Additional file 3: Figure S3 and Additional file 4: Figure. S4). It may indicate that the enhanced virion replication happens at low expression levels of these other genes.

## Conclusions

Compared with the samples singly infected with SRBSDV or RRSV, the viral movement protein and viroplasm matrix-related genes as well as the structural (capsid) protein genes of the two viruses were remarkably up-regulated bilaterally at different time points in the co-infected rice plants, while the RNA silencing suppressor (P6) of only RRSV, but not of both the viruses, was up-regulated. We therefore proposed that the SRBSDV-RRSV synergism promoted replication and movement of both the viruses and inhibited the host immunity by enhancing the gene suppressing effect exerted by one of them (RRSV), leading to an increase of both viruses’ titers in the co-infected rice plants.

## Additional files

Additional file 1: Figure S1. The expression levels of structural protein genes of SRBSDV (P1 to P4, P8 and P10) at 9, 15 and 20 dpi in SRBSDV-infected and co-infected rice. Besides P10, which was significantly up-regulated (Fig. 4), the remaining genes were slightly up-regulated at 9, 15 and 20 dpi in co-infected plants. Vertical bars correspond to error bars indicating standard deviation (Bonferroni’s honestly significant difference test, *P* = 0.05). (JPEG 300 kb)

Additional file 2: Figure S2. The expression levels of structural protein genes of RRSV (P1 to P5, P8 and P9) at 9, 15 and 20 dpi in RRSV-infected and co-infected rice. Besides P3 at 15 and 20 dpi (Fig. 2), and P8 at 9, 15 and 20 dpi (Fig. 4), which were significantly up-regulated, the remaining genes were slightly up-regulated at 9, 15 and 20 dpi in co-infected plants. Vertical bars correspond to error bars indicating standard deviation (Bonferroni’s honestly significant difference test, *P* = 0.05). (JPEG 309 kb)

Additional file 3: Figure S3. The expression levels of non-structural protein genes of SRBSDV (P5-1, P5-2, P7-1, P7-2, P9-1 and P9-2) at 9, 15 and 20 dpi in SRBSDV-infected and co-infected rice. Besides P5-1 at 15 dpi (Fig. 1), P7-1 at 15 and 20 dpi (Fig. 3) and P9-1 at 9, 15 and 20 dpi (Fig. 1), which were significantly up-regulated, the remaining genes were slightly up-regulated at 9, 15 and 20 dpi in co-infected plants. Vertical bars correspond to error bars indicating standard deviation (Bonferroni’s honestly significant difference test, *P* = 0.05). (JPEG 225 kb)

Additional file 4: Figure S4. The expression levels of non-structural protein genes of RRSV (P6, P7 and P10) at 9, 15 and 20 dpi in RRSV-infected and co-infected rice. P7 was slightly up-regulated at 9, 15 and 20 dpi, while P6 at 9, 15 and 20 dpi, and P10 at 15 dpi (Fig. 2) were significantly up-regulated in co-infected plants. Vertical bars correspond to error bars indicating standard deviation (Bonferroni’s honestly significant difference test, *P* = 0.05). (JPEG 203 kb)

## Abbreviations

BPH: Brown planthopper
CP: Capsid protein
Dpi: Days post inoculation
dsRNA: double stranded RNA
MP: Movement protein
ORF: Open reading frame
RdRp: RNA-dependent RNA polymerase
RRSV: Rice ragged stunt virus
RSS: RNA silencing suppressor
RT-qPCR: Reverse transcription-quantitative PCR
SRBSDV: Southern rice black-streaked dwarf virus
WBPH: White-backed planthopper
