# Supplementary figures and images for: Co-infection of two reoviruses increases both viruses accumulation in rice by up-regulating of viroplasm components and movement proteins bilaterally and RNA silencing suppressor unilaterally
Source: Virol J. 2017 Aug 8;14:150. doi: 10.1186/s12985-017-0819-0 (PMC5549333; doi:10.1186/s12985-017-0819-0)

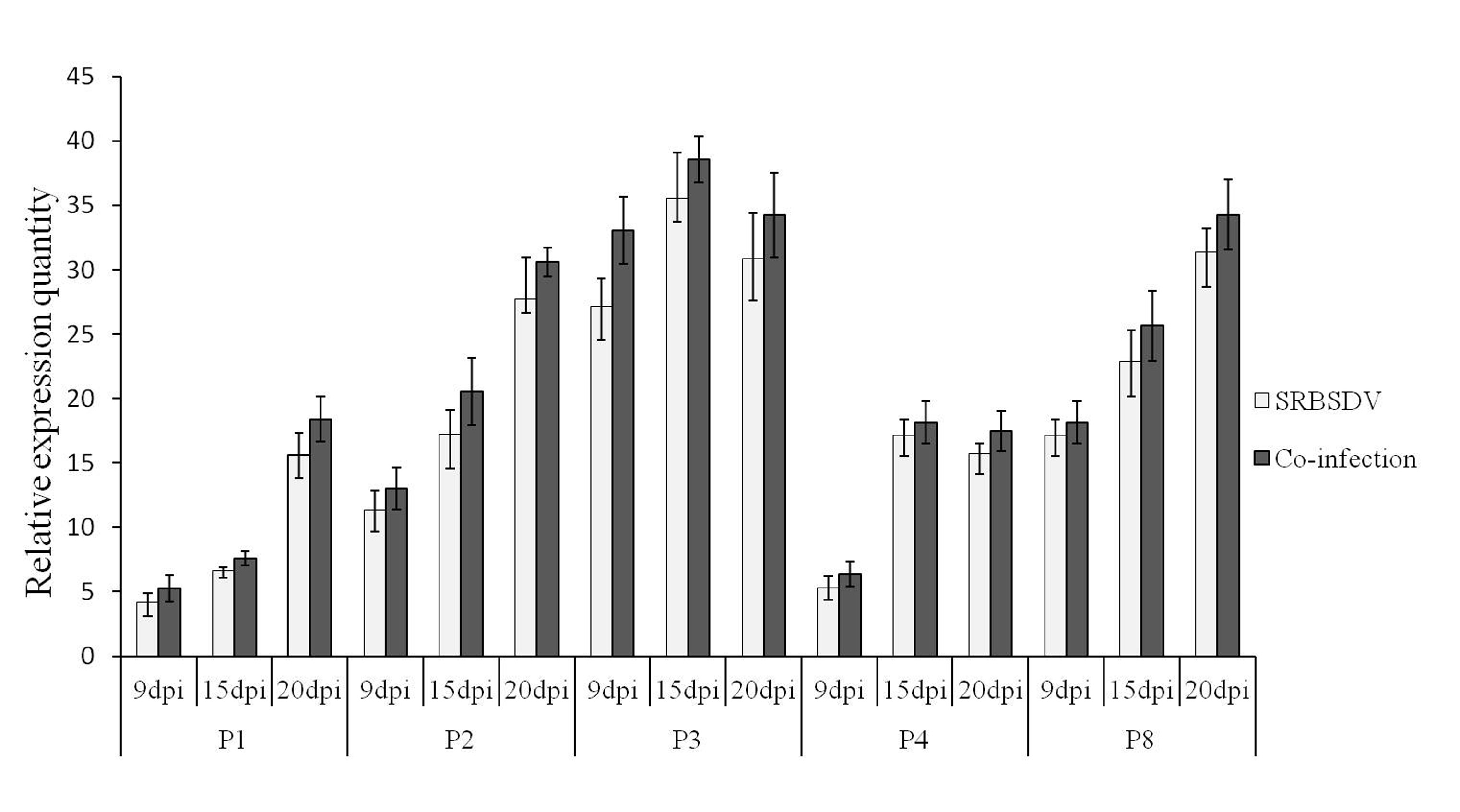

Supplement: Supplementary file 1 — The expression levels of structural protein genes of SRBSDV (P1 to P4, P8 and P10) at 9, 15 and 20 dpi in SRBSDV-infected and co-infected rice. Besides P10, which was significantly up-regulated (Fig. 4), the remaining genes were slightly up-regulated at 9, 15 and 20 dpi in co-infected plants. Vertical bars correspond to error bars indicating standard deviation (Bonferroni’s honestly significant difference test, P = 0.05). (JPEG 300 kb) [file 12985_2017_819_MOESM1_ESM.jpg]

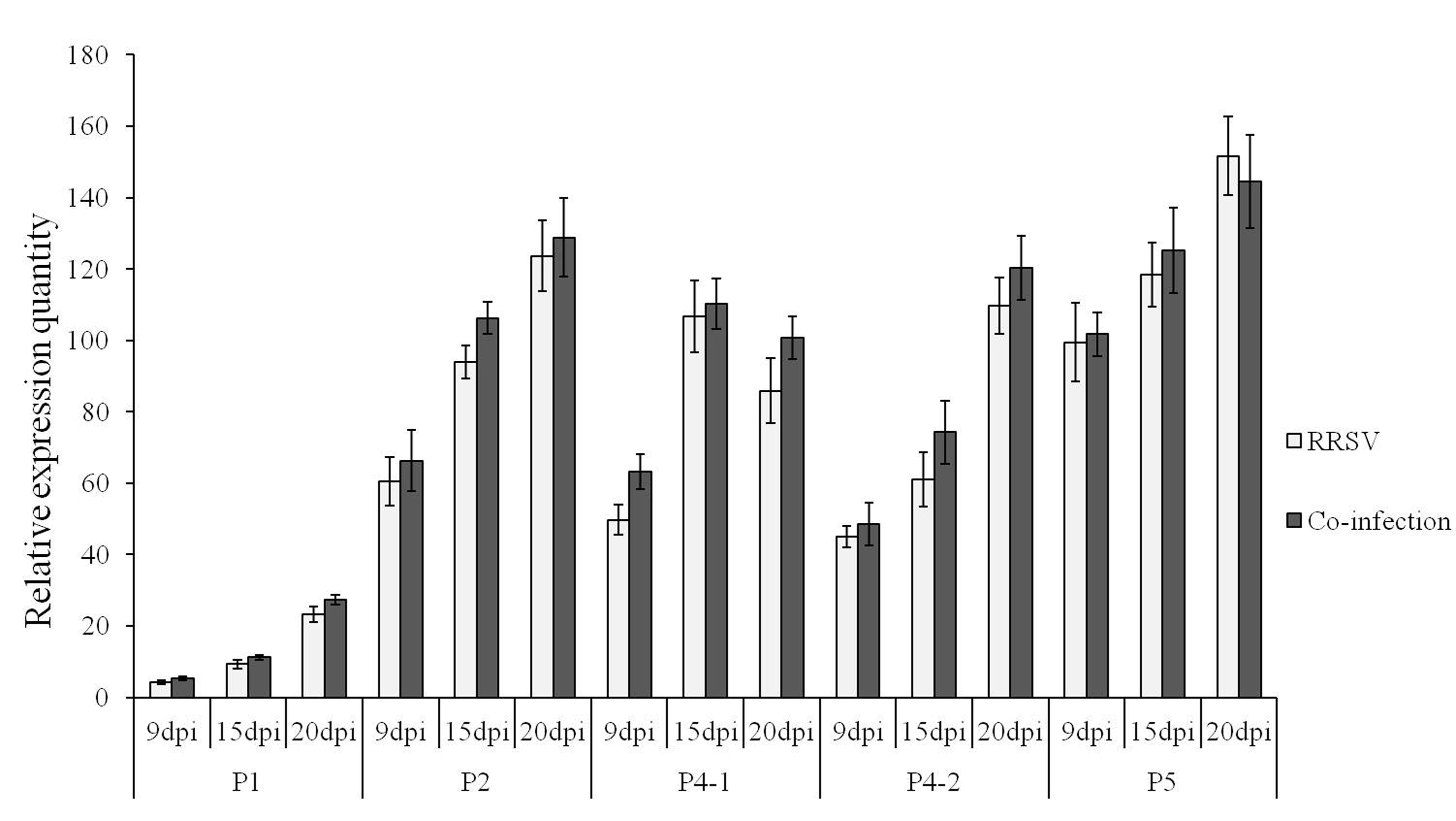

Supplement: Supplementary file 2 — The expression levels of structural protein genes of RRSV (P1 to P5, P8 and P9) at 9, 15 and 20 dpi in RRSV-infected and co-infected rice. Besides P3 at 15 and 20 dpi (Fig. 2), and P8 at 9, 15 and 20 dpi (Fig. 4), which were significantly up-regulated, the remaining genes were slightly up-regulated at 9, 15 and 20 dpi in co-infected plants. Vertical bars correspond to error bars indicating standard deviation (Bonferroni’s honestly significant difference test, P = 0.05). (JPEG 309 kb) [file 12985_2017_819_MOESM2_ESM.jpg]

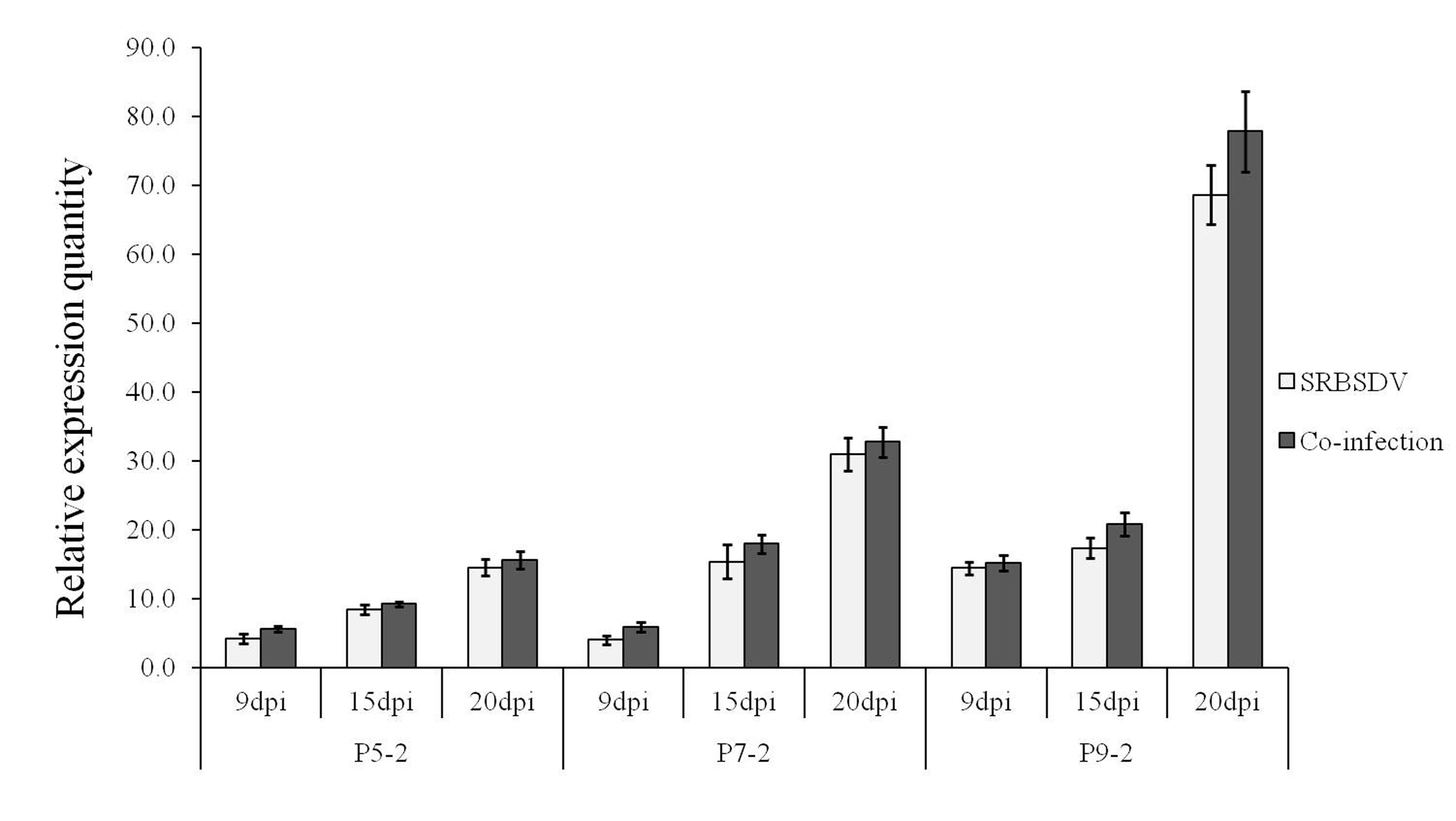

Supplement: Supplementary file 3 — The expression levels of non-structural protein genes of SRBSDV (P5-1, P5-2, P7-1, P7-2, P9-1 and P9-2) at 9, 15 and 20 dpi in SRBSDV-infected and co-infected rice. Besides P5-1 at 15 dpi (Fig. 1), P7-1 at 15 and 20 dpi (Fig. 3) and P9-1 at 9, 15 and 20 dpi (Fig. 1), which were significantly up-regulated, the remaining genes were slightly up-regulated at 9, 15 and 20 dpi in co-infected plants. Vertical bars correspond to error bars indicating standard deviation (Bonferroni’s honestly significant difference test, P = 0.05). (JPEG 225 kb) [file 12985_2017_819_MOESM3_ESM.jpg]

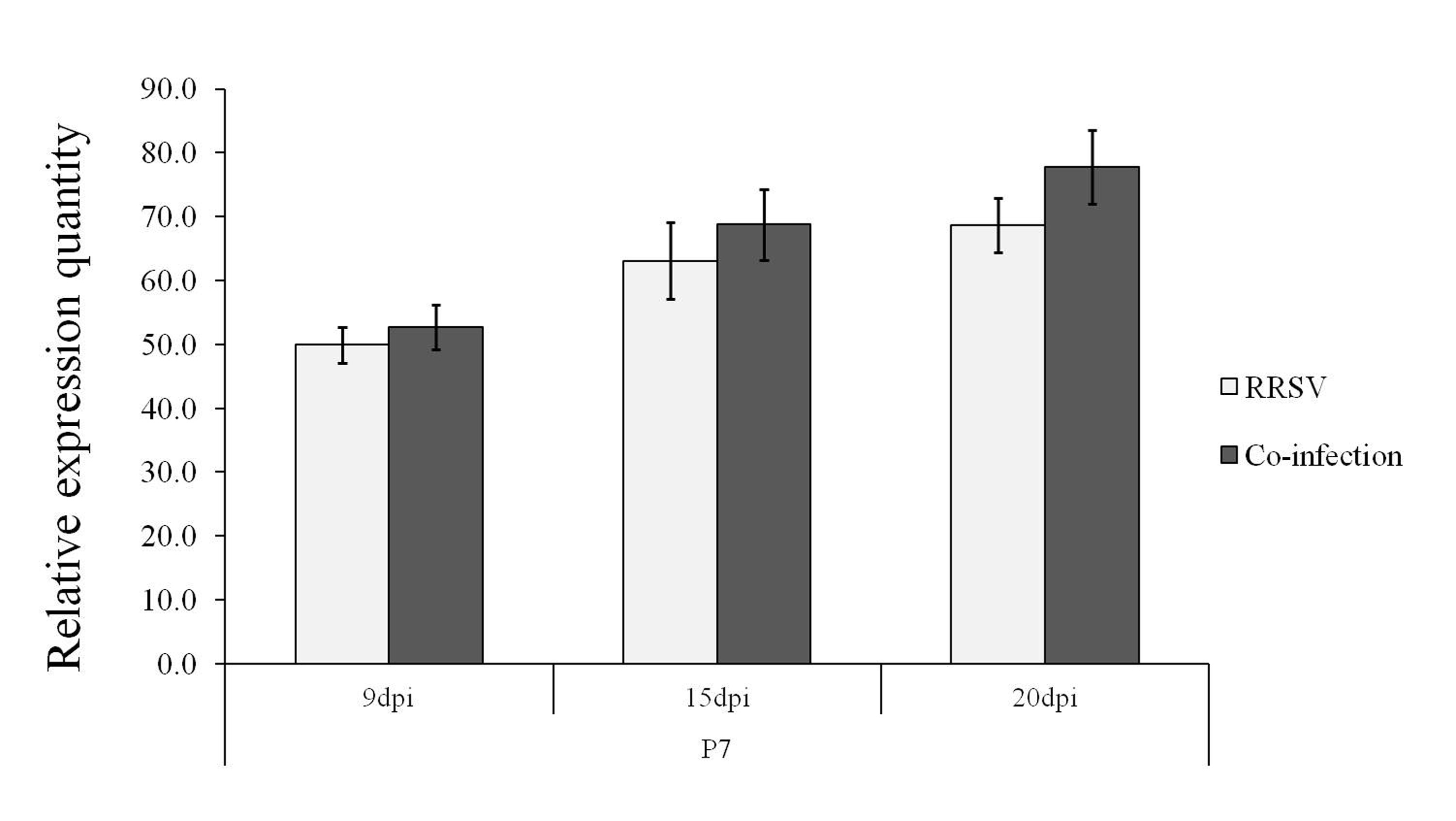

Supplement: Supplementary file 4 — The expression levels of non-structural protein genes of RRSV (P6, P7 and P10) at 9, 15 and 20 dpi in RRSV-infected and co-infected rice. P7 was slightly up-regulated at 9, 15 and 20 dpi, while P6 at 9, 15 and 20 dpi, and P10 at 15 dpi (Fig. 2) were significantly up-regulated in co-infected plants. Vertical bars correspond to error bars indicating standard deviation (Bonferroni’s honestly significant difference test, P = 0.05). (JPEG 203 kb) [file 12985_2017_819_MOESM4_ESM.jpg]
